# Skin malformations in a neonatal foal tested homozygous positive for Warmblood Fragile Foal Syndrome

**DOI:** 10.1186/s12917-015-0318-8

**Published:** 2015-01-31

**Authors:** Chloé Monthoux, Simone de Brot, Michelle Jackson, Ulrich Bleul, Jasmin Walter

**Affiliations:** Clinic of Reproductive Medicine, Vetsuisse Faculty, University of Zurich, Winterthurerstrasse 260, 8057 Zurich, Switzerland; Institute of Veterinary Pathology, Vetsuisse Faculty, University of Zurich, Winterthurerstrasse 260, 8057 Zurich, Switzerland; Current address: School of Veterinary Medicine and Science, University of Nottingham, Sutton Bonington, LE12 5RD UK; Equine Department, Section Surgery, Vetsuisse Faculty, University of Zurich, Winterthurerstrasse 260, 8057 Zurich, Switzerland

**Keywords:** PLOD1, Equine procollagen-lysine, 2-oxoglutarate 5-dioxygenase 1, LH1, Lysyl hydroxylase 1, Ehlers-Danlos syndrome, Dermatosparaxis, Dermal hyperfragility, Horse, Equine, Connective tissue, Hereditary

## Abstract

**Background:**

Skin malformations that resembled manifestations of Ehlers-Danlos-Syndrome were described in a variety of domestic animals during the last century as cutis hyperelastica, hyperelastosis cutis, dermatosparaxis, dermal/collagen dysplasia, dermal/cutaneous asthenia or Ehlers-Danlos-like syndrome/s. In 2007, the mutation responsible for Hereditary Equine Regional Dermal Asthenia (HERDA) in Quarter Horses was discovered. Several case reports are available for similar malformations in other breeds than Quarter Horses (Draught Horses, Arabians, and Thoroughbreds) including four case reports for Warmblood horses. Since 2013, a genetic test for the Warmblood Fragile Foal Syndrome Type 1 (WFFS), interrogating the causative point mutation in the equine *procollagen-lysine, 2-oxoglutarate 5-dioxygenase 1* (PLOD1, or lysyl hydroxylase 1) gene, has become available. Only limited data are available on the occurrence rate and clinical characteristics of this newly detected genetic disease in horses. In humans mutations in this gene are associated with Ehlers-Danlos Syndrome Type VI (kyphoscoliotic form).

**Case presentation:**

This is the first report describing the clinical and histopathological findings in a foal confirmed to be homozygous positive for WFFS. The Warmblood filly was born with very thin, friable skin, skin lesions on the legs and the head, and an open abdomen. These abnormalities required euthanasia just after delivery. Histologic examination revealed abnormally thin dermis, markedly reduced amounts of dermal collagen bundles, with loosely orientation and abnormally large spaces between deep dermal fibers.

**Conclusion:**

WFFS is a novel genetic disease in horses and should be considered in cases of abortion, stillbirth, skin lesions and malformations of the skin in neonatal foals. Genetic testing of suspicious cases will contribute to evaluate the frequency of occurrence of clinical WFFS cases and its relevance for the horse population.

## Background

A hereditary disease with symptoms of hyperextensible and abnormally fragile skin, as well as hyperextensibility of articulations, was first described in humans in 1892 [1]. The name Ehlers-Danlos Syndrome (EDS) was proposed in 1934 to describe this disease complex [2]. This syndrome is caused by various mutations with different modes of heredity and was categorized into different subtypes because of variation in affection of blood vessels, internal organs, wound healing and impact on the pregnancy, additional to the typical lesions on skin and joints [3-5].

EDS-like diseases have been described for decades in cat [6], dog [7], rabbit [8], mink [7], cattle [9], sheep [10] and horse [9,11], with variable heredity in the different species. These syndromes were commonly described as cutis hyperelastica, hyperelastosis cutis, dermatosparaxis, dermal/collagen dysplasia, dermal/cutaneous asthenia or Ehlers-Danlos-like syndrome/s [8,9,11-17].

« Ehlers-Danlos-like syndromes » (EDLS) will be used in this paper to describe the syndromes in animals, in which the clinical findings are similar to human EDS, independent of their origin.

In several cases of EDLS in different species, mutations were detected in genes encoding for enzymes involved in production of collagen or other components of the dermal extracellular matrix [2,12,13,18-22].

In equines, EDLS are described in Draught horses [14], Warmblood horses [9,15,23,24], Arabians [25], Quarter Horses and Quarter lineage [16,26-37] as well as Thoroughbreds [11]. These syndromes were described in neonates, juveniles and adults. Two gene defects causing an EDLS phenotype in horses have been discovered in recent years. Since 2007, the test for the well documented hereditary equine regional dermal asthenia (HERDA) in Quarter Horses and related breeds, testing for the known PPIB:p.39Gly > Arg mutation of the peptidylprolyl isomerase B (cyclophilin B) gene, is available [13,16,31-36,38].

For Warmblood Fragile Foal Syndrome Type 1 (WFFS), a genetic test developed by N. Winand became commercially available in 2013^a^ [39]. This autosomal recessive inherited disease occurs in Warmblood horses and related breeds and is reportedly caused by a point mutation *(*c.2032G > A, p.Gly678Arg*)* in the equine procollagen-lysine, 2-oxoglutarate 5-dioxygenase 1 (*PLOD1*) gene [39].

PLOD1 is an important posttranslational modifying enzyme in collagen biosynthesis, which hydroxylates specific lysines in collagen [19]. These hydroxylysines act as precursors for crosslinking that are responsible for the tensile strength, mechanical stability of collagen fibrils and are involved in the formation of fibers [4,19,21]. *Plod1* knock-out mice are flaccid, have gait abnormalities and about 15% of them die because of aortic rupture. Smooth muscle cells in non-ruptured *Plod1*^*−/−*^ aortas showed degenerative changes and collagen fibrils in the *Plod1*^*−/−*^ aorta and skin showed abnormal morphology [40]. Over 20 different mutations have been reported for the human *PLOD1* gene and are associated with clinical Ehler-Danlos-Syndrome type VI, also called the kyphoscoliotic type, with the most common human mutation (25%) being a duplication of exons 10–16 [19-21,41]. Ten human pathogenic variants were point mutations in different exon regions of PLOD1, which were also shown to be associated with a decreased enzyme activity in Ehler-Danlos Type VI patients [21]. Symptoms of this autosomal recessive inherited connective tissue fragility syndrome occur from birth on and are characterized by skin hypersensibility, muscle hypotonia, joint laxity, eyes and vessels involvement, scoliosis and premature rupture of fetal membranes [3,4,18-21,41-43]. Life span of these patients may be normal, but affected individuals are at risk for rupture of medium sized arteries [4,19].

The decreased activity of PLOD1 leads to reduced hydroxylysine-based pyridinoline cross-links in collagens and therefore to an increased lysylpyridinoline (LP) to hydroxylysylpyridinoline (HP) ratio in urine, which is used to diagnose kyphoscoliotic EDS VI [4]. Another method to test these patients for EDS, kyphoscoliotic form, is the measurement of PLOD1 enzyme activity in cultured fibroblasts [4,19].

Mutations in the *PLOD1* gene were not described in animals up to the patent application for the WFFS test [39]. To our knowledge, this is the first case report describing the clinical and histopathological changes in a genetically confirmed homozygous WFFS foal*.*

## Case presentation

**Figure 1:**
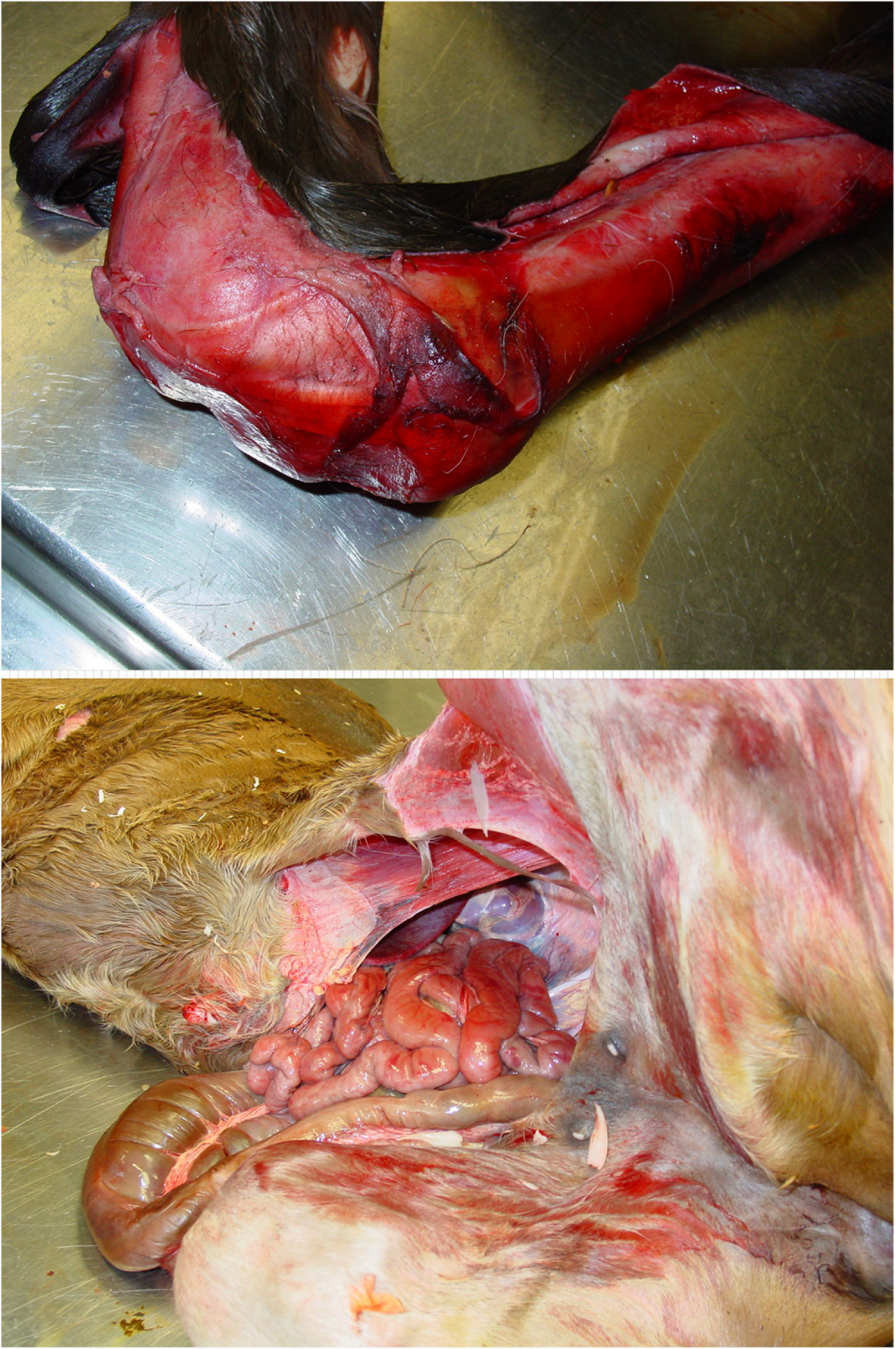
**Clinical presentation of the WFFS foal.** The foal presented with severe ablation of skin on the right front leg (above) and an open abdomen and eventration of the small intestines (below).

A 5-year-old Westfalian Warmblood mare was referred to the Clinic of Reproductive Medicine for dystocia in March 2012. During pregnancy, no complications were detected and parturition occurred near the calculated date of term. The obstetrical examination revealed a living foal in cranial presentation, dorso-sacral position, with bilateral carpal flexion. Correction of carpal flexion was not possible in the standing mare, resulting in the decision to perform a controlled vaginal delivery under general anaesthesia. Ten minutes after induction of anaesthesia, correction of bilateral carpal flexion was possible after elevation of the mare’s hindquarters. A living filly was extracted after solely manual correction with slight traction. The foal presented with an open abdomen and extensive skin lesions (Figure 1). Obstetrical devices were not used for the gentle correction and extraction procedure, which makes idiopathic causes for the injuries unlikely. The newborn was euthanized due to poor prognosis and a full necropsy was performed. The mare was released from the clinic in healthy condition after treatment for retained foetal membranes.

### Pathological findings

On macroscopic necropsy examination, the foal presented with multiple extensive (up to 20 cm in length) and severe skin lacerations on both front legs and face. The abdomen was open on midline measuring approximately 30 cm in length with eventration of the small intestines.

**Figure 2:**
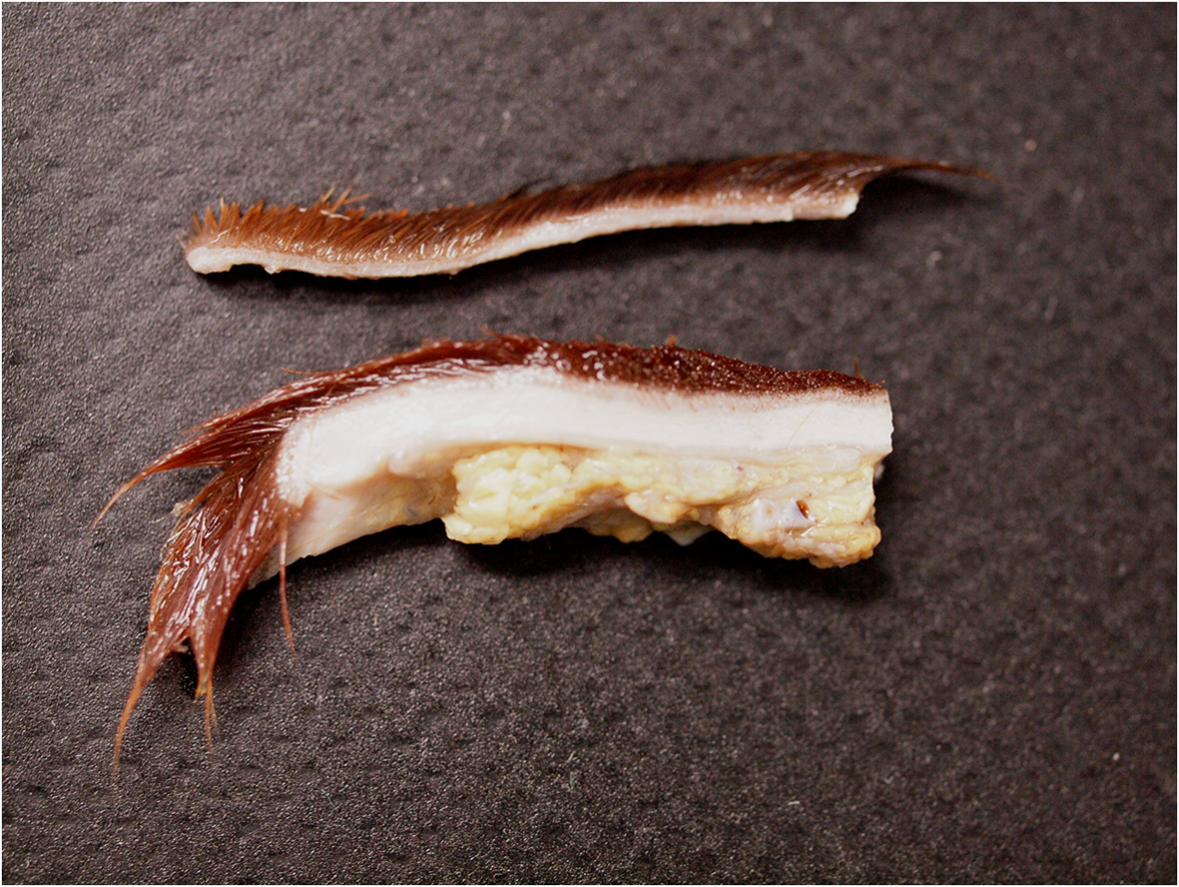
**Skin morphology of the WFFS foal.** Affected skin from the front limb (above) is markedly thinner (<1.5 mm) than unaffected skin from the back (below) of the same foal.

In the affected areas, the skin was extremely fragile and thin (1 to 2 mm), friable and very loosely attached to the underlying subcutaneous tissue (Figure 2). Pressure points on head as well as front and hind limbs showed subcutaneous oedema, hematomas and seromas, particulary affected were the fetlocks and tarsus regions.

**Figure 3:**
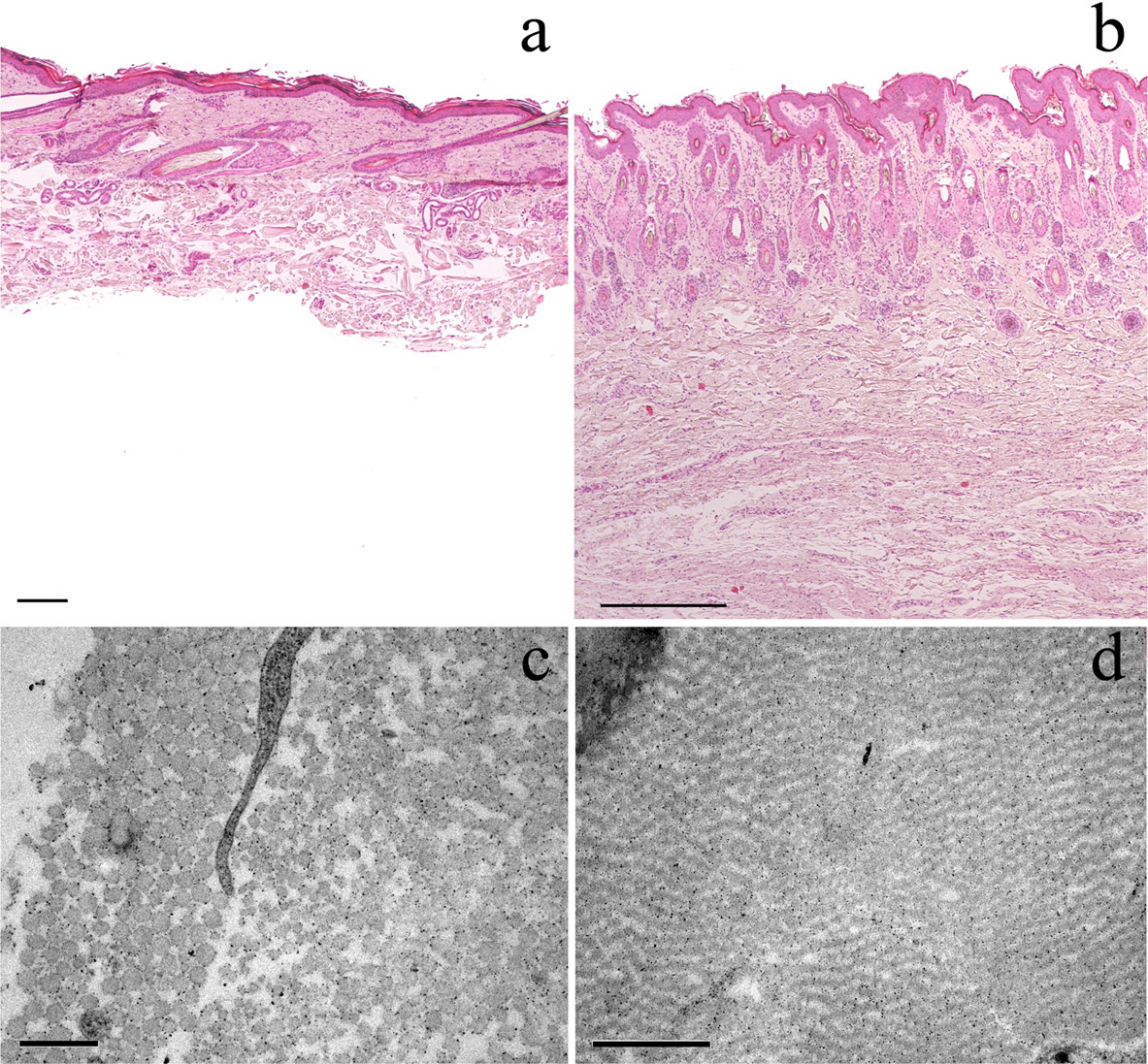
**Light and electron microscopy of affected (a&c) and unaffected (b&d) skin.** Light microscopy of affected **(a)** and unaffected **(b)** skin: The deep dermis is markedly thinned (approximately 30% of dermal thickness of hind limb skin of unaffected 1-day-old control Warmblood foal **(b)**) and shows a reduced number of thin, irregular collagen bundles separated by clear spaces. The epidermis and adnexal structures appear normal. Subcutaneous tissue was detached from the overlying dermis and is missing on this slide. The size bars indicate 200 μm. Haematoxylin and eosin staining (HE), 4x. Electron microscopy of affected **(c)** and unaffected **(d)** skin: In the affected skin **(c)** (front limb) a mild randomness in orientation and variability of the cross-sections of the collagen fibrils are present. The size bar indicates 0.5 μm. The skin of the control **(d)** (hind limb from a 1-day-old control Warmblood foal) shows regular orientation of collagen fibrils with no visible variability of cross-sections. The size bar indicates 1 μm.

Several samples of lesional and non-lesional skin were fixed in 10% neutral buffered formalin, paraffin-embedded, sectioned at 3 μm, and stained with haematoxylin and eosin (HE) for light microscopy. On histologic examination, the abnormally thin dermis showed a markedly reduced amount of dermal collagen bundles that were of variable thickness and orientation and loosely arranged with abnormally large spaces between deep dermal fibers. The epidermis and adnexal structures were normal (Figure 3).

Electron microscopy (EM) was performed on affected skin samples from various locations of the body. For negative contrast EM, selected tissues were fixed in 2.5% glutaraldehyde and 1% osmium tetroxide (OsO4) and buffered with 0.1 M sodium phosphate (pH 7.4). After fixation, the tissues were dehydrated in an ethanol series, embedded in Epoxy resin^b^, cut in ultrathin (90 nm) sections and stained with uranyl acetate and lead citrate.

EM examination revealed only mild abnormalities. The collagen fibrils were reasonably well organised in bundles forming parallel arrays and showed regular periodic banding. However, mild randomness in orientation and mild variability of the cross-sections of the collagen fibrils were present (Figure 3).

### Genetic tests

**Figure 4:**
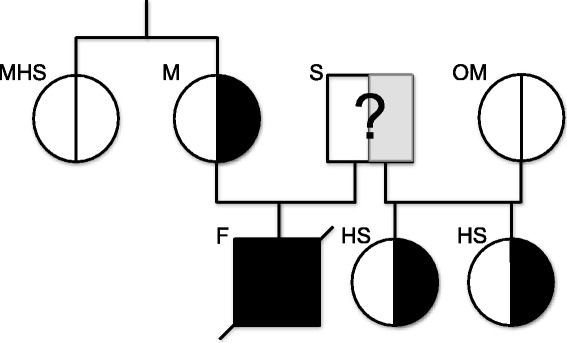
**Results of the genetic testing of the foal and close relatives.** WFFS carriers in the relatives of the homozygous foal are the mare (M) and two of his half-sisters (HS) from the same stallion (S). The mothers of the half-sisters (OM) and the mare’s half-sister (MHS) were homozygous for the wild-type allele. The stallion was not tested for WFFS.

At the time of necropsy, the only available genetic test for EDLS was the test for HERDA, for which the mare was homozygous negative. In spring 2013, when the WFFS-test became commercially available^a^, the mare was tested and resulted heterozygous positive for the WFFS allele (*A/G*). The stallion was not tested. Consequently, formalin fixed muscle tissue of the foal was also submitted for genetic testing. Genomic DNA was extracted from muscle tissue using the commercially available MagNA Pure 96 DNA and Viral NA Small Volume Kit^c^ following manufacturers’ instructions. Genotyping was performed for the XM_001491331:c.2032G > A variant in the *PLOD1* gene, which is supposed to be the causative variant for Warmblood Fragile Foal Syndrome type 1 (WFFS) according to the patent application [39] (primers and PCR conditions available from Laboklin, Bad Kissingen, Germany upon request). The results revealed a homozygous positive WFFS *(A/A)* genotype for the foal. Close relatives to the foal, all healthy, were also tested for the WFFS genotype and revealed either a carrier genotype (*A/G*; two tested half-sisters of the foal sired by the same stallion) or a homozygous wild type genotype (*G/G*; two mothers of the heterozygous half-sisters and the full sister of the mother of the homozygous positive and symptomatic foal) (Figure 4). All samples for genetic analysis were submitted to the laboratory without mentioning clinical suspicion for WFFS. The Clinic of Reproductive Medicine and the owner of the horses were fully charged for all tests.

## Discussion

Case reports about EDLS describing clinical, histological and EM findings in horses were well recorded this past decade, but usually without any genetic confirmation as HERDA and WFFS tests were not available. Now, after the discovery of two genetic causes, previous cases may be explained by the newly detected mutations.

The majority of published EDLS case reports in horses describe the clinical signs of HERDA mutation in the Quarter Horse. Nevertheless, there are several EDLS cases documented in other breeds than Warmblood or Quarter horses [11,14,25]. These horses presented with several of the following symptoms: skin hyperelasticity; loss of the hoof capsule; lesions on the back, the flanks, shoulders and legs; pathologic scar formation; and abortion or embryonic loss in related pairings [11,14,25].

Literature of HERDA homozygous symptomatic horses, after the launch of the genetic test in 2007 [13,31-33], enabled the description of a more precise phenotype for this mutation: fragile and hyperextensible skin, lacerations, seromas, haematomas, ulcerations and scars with typical localisation on the dorsal region extending to other parts of the body [16]. The disease may also affect other tissues, such as tendons, ligaments, great vessels or the cornea. These clinical signs most commonly emerge in older horses; however, documentation of neonatal cases exist [16].

Genetically confirmed WFFS cases were not available in previous literature; however, four case reports of EDLS clinical signs in Warmblood horses have been reported [9,15,23,24]. The first two cases in neonates (one and six days old) showed skin lesions on the limbs, abdominal region, withers and underneath the tail, with hyperextension of the limb articulations, hydrops, subcutaneous emphysema, and a general modified integument [9,23]. In another case report, a 6-week-old foal developed haematomas, multiple lacerations, scars and oedema and displayed poor wound healing with skin lesions localised on the flank, stifle and limb regions [15]. Regarding age (1.5 years) and localisation of lesions, the last case is atypical. In this case, the horse displayed wound-healing problems and dermatological modifications of the skin and hair on the flank and back. The dorsal skin, lateral to the thoracolumbar region, was hyperelastic and painful [24]. Retrospectively, these four clinical cases without association to a specific genotype are now suspicious for WFFS. As suggested by Steelman (2013), EDLS in horses seems to be a collection of similar phenotypes with different genetic origin [36], so differentiation between HERDA, WFFS and other EDLS cases is difficult without genetic testing (Table 1).

**Table 1:** **Synoptical table for congenital skin lesions and malformations in horses**

**Disease**	**WFFS**	**HERDA**	**EDLS**
**Full Name**	Warmblood Fragile Foal Syndrome	Hereditary Equine Regional Dermal Asthenia	Ehlers-Danlos-like syndrome of unknown origin
**Breed**	Warmblood	Quarter Horse (lineage: stallion Poco Bueno)	Warmblood, Quarter horse, Drafthorse, Arabian, Thoroughbred
**Gene**	*PLOD1:* “procollagen-lysine, 2-oxoglutarate 5-dioxygenase 1” (LH1: lysyl hydrohylase 1)	*PPIB:* “peptidyl-prolyl cis-trans isomerase B” (Cyclophilin B)	Involved genes not elucidated up to now
**Symptoms**	Lesions of skin/mucosa (tearing, ulcerations); Hyperextension of articulations, floppy ears, hydrops, subcutaneous emphysema, hematomas, premature birth	Fragile and hyper extensible skin, lacerations, seromas, hematomas, ulcerations, scars	Skin hyper elasticity, loss of hoof capsule, lesions on back, flanks, shoulders, legs, scarring, pregnancy loss, retention of fetal membranes
**Localisation of lesions**	Head, neck, thorax, legs, abdomen, stifle, withers, perineum, oral mucous membranes, articular cartilage	Back and from back extending, may involve: tendons, ligaments, great vessels, cornea	Variable
**Age**	Most common in neonates	Mostly older horses, also possible in neonates	Neonates as well as adult horses
**Heredity**	Autosomal recessive	Autosomal recessive	Heredity mode not described
**Genetic testing available**	Yes	Yes	No
**Prevalence of mutation**	Up to 11.11% in clinically normal adult Warmblood horses	Prevalence up to 28.3% in Cutting horses	Prevalence unknown
**Data sources**	Patent application	Ishikawa et al. (2012) [13]	Gunson et al. (1984) [25]
	Winand N (2012)	Mochal et al. (2010) [33]	Esser M. et al. (1999) [19]
		Grady et al. (2009) [32]	Solomons B (1984) [11], Rufenacht S et al. (2010) [24]
			Steelman et al. (2013) [36]

Necropsy alone appears insufficient to diagnose one of the EDLS types, as many similarities were observed in the lesions of our positive tested WFFS case, the cases retrospectively suspicious for WFFS and other documented EDLS cases. Additionally, it is unclear if other atypical phenotypes exist for this disease. Perhaps the birth of foals with cutaneous malformations is not the only clinical presentation. In humans, EDS symptomatic fetuses of asymptomatic mothers were affected by premature birth, still birth and abortion [18,42-47]. As the foetal membranes find their origin in the embryo, the connective tissue in these membranes may also be affected and leading to their premature rupture, causing the above described complications of pregnancy [44]. These clinical manifestations of cases with similar causative mechanism can lead to the suggestion that cases of abortion, still birth and premature labour may also be caused by WFFS, even when there are no case reports available. Widespread genetic testing, especially of abortions and symptomatic offspring of heterozygous (A/G) pairings, will help to obtain a more detailed description of the clinical signs that may be caused by this mutation.

Detailed information about the prevalence of the WFFS mutation is not available in the literature. The human EDSVI is rare with a disease incidence of approximately 1:100,000 live births and an estimated carrier frequency of 1:150 [19,20]. The patent application for the test describes test results of 124 horses. The carrier frequency in the tested clinically normal adult Warmblood population was 11.11%. Perfect association between homozygosity (*A/A*, n = 2) and clinical WFFS symptoms was observed. Heterozygous (*A/G*, n = 8) and wild type homozygous (*G/G*, n = 114) animals were all phenotypically normal. The carrier frequency in the initial test population was corroborated by the data of 500 randomly selected Warmblood horses in Germany, where 9.5% of the population were detected as carriers (Personal Communication: Gunreben B.^a^; 2013). The seven horses tested for this case report reflect these results with regard to association between genotype and phenotype: a clinical phenotype was observed solely in the homozygous positive foal. Up to now, no reports are available for genetically confirmed homozygous positive animals for the WFFS mutation with absence of clinical symptoms.

## Conclusions

Due to the severe clinical signs, this novel genetic disease requires further investigation to obtain robust data on prevalence of the mutation as well as the correlation between genotype and phenotype. Homozygous (*A/A*) offspring can be avoided from now on easily by breeding heterozygous carriers only to homozygous negative breeding partners. Awareness for this new disease will hopefully result in the genetic testing of suspicious cases as identification of homozygous animals provides the unique opportunity to study the biochemical and clinical consequences of the mutation to obtain an in-depth characterization of WFFS.

## Endnotes

*Manufacturers’ details*

^a^Laboklin GmbH&Co.KG, Bad Kissingen, Germany

^b^Epon, Fluka, Sigma-Aldrich, St-Louis, MO, USA

^c^Roche Diagnostics International AG, Rotkreuz, Switzerland.

## Abbreviations

EDS: Ehlers-Danlos syndrome
EDLS: Ehlers-Danlos-like syndromes
EDSVI: Ehlers-Danlos syndrome type VI
HERDA: Hereditary equine regional dermal asthenia
LH1: Lysyl hydroxylase 1
PLOD1: Procollagen-lysine, 2-oxoglutarate 5-dioxygenase 1
PPIB: Peptidylprolyl isomerase B (cyclophilin B)
WFFS: Warmblood fragile foal syndrome type 1
